# The effects of a three-week use of lumbosacral orthoses on trunk muscle activity and on the muscular response to trunk perturbations

**DOI:** 10.1186/1471-2474-11-154

**Published:** 2010-07-07

**Authors:** Jacek Cholewicki, Kevin C McGill, Krupal R Shah, Angela S Lee

**Affiliations:** 1 Department of Surgical Specialties, College of Osteopathic Medicine, Michigan State University, East Lansing, MI, USA; 2 Feinberg School of Medicine, Northwestern University, Chicago, IL, USA; 3 Department of Internal Medicine and Pediatrics, University of Michigan, Ann Arbor, MI, USA

## Abstract

**Background:**

The effects of lumbosacral orthoses (LSOs) on neuromuscular control of the trunk are not known. There is a concern that wearing LSOs for a long period may adversely alter muscle control, making individuals more susceptible to injury if they discontinue wearing the LSOs. The purpose of this study was to document neuromuscular changes in healthy subjects during a 3-week period while they regularly wore a LSO.

**Methods:**

Fourteen subjects wore LSOs 3 hrs a day for 3 weeks. Trunk muscle activity prior to and following a quick force release (trunk perturbation) was measured with EMG in 3 sessions on days 0, 7, and 21. A longitudinal, repeated-measures, factorial design was used. Muscle reflex response to trunk perturbations, spine compression force, as well as effective trunk stiffness and damping were dependent variables. The LSO, direction of perturbation, and testing session were the independent variables.

**Results:**

The LSO significantly (*P *< 0.001) increased the effective trunk stiffness by 160 Nm/rad (27%) across all directions and testing sessions. The number of antagonist muscles that responded with an onset activity was significantly reduced after 7 days of wearing the LSO, but this difference disappeared on day 21 and is likely not clinically relevant. The average number of agonist muscles switching off following the quick force release was significantly greater with the LSO, compared to without the LSO (*P *= 0.003).

**Conclusions:**

The LSO increased trunk stiffness and resulted in a greater number of agonist muscles shutting-off in response to a quick force release. However, these effects did not result in detrimental changes to the neuromuscular function of trunk muscles after 3 weeks of wearing a LSO 3 hours a day by healthy subjects.

## Background

Abdominal belts and lumbosacral orthoses (LSOs) are designed to provide support to the lumbar spine. Abdominal belts are used in ergonomics to prevent low back injury, while orthoses are used in clinical settings for conservative and postsurgical management of low back pain (LBP). Both of these devices function in a biomechanically similar manner by reducing trunk range of motion [1-4] and increasing trunk stiffness [5-8]. Anecdotal evidence suggests that people feel "safer" and "more stable" during physical exertions when wearing abdominal belts. In self-reported surveys, people with LBP report that wearing a LSO allows them to continue their activities with less discomfort [9-12]. Though a recent, multi-center, randomized, clinical study showed that LSOs significantly improved functional status, pain level, and analgesic medication use in patients with LBP [13], there is still a preponderance of evidence challenging the use of lumbar supports to prevent injury or relieve LBP [3,14-18]. A systematic review within the framework of Cochrane Database [3,14-18] concluded that there was no evidence that lumbar supports are effective in preventing LBP, but it remains unclear whether they are effective in the treatment of LBP. However, the authors noted that the overall methodological quality of the studies in this review was rather low. One of the most frequent threats to validity was low patient compliance.

Despite the controversy about the effectiveness of LSOs, they are commonly used in clinical settings [19,20]. With their use, a growing concern arises that LSOs weaken trunk muscles and increase patients' susceptibility to injury after they discontinue using LSOs [20,21]. This concern stems from the belief that LSOs support the lumbar spine in a way that reduces the work demand on trunk muscles. However, this effect has not been demonstrated quantitatively. Studies that carefully controlled posture and trunk kinematics did not find any significant reduction in muscle activity or spine compression force due to a LSO [3,22,23]. There was also no evidence of muscle weakening in individuals with LBP or healthy controls after a period of wearing a LSO [24-26].

However, there is a possibility that subtle changes in trunk muscle control, heretofore unmeasured, take place when a LSO is worn. For example, an increased injury incidence rate was reported in a large study of airline luggage handlers who started and later discontinued wearing an abdominal belt [27]. This study suggested that subtle neuromuscular adaptation may have occurred during the initial use of lumbar supports, which may have predisposed the workers to injury after they discontinued their use and no longer had the benefit of the abdominal belt's passive support [27]. We hypothesized previously that passively augmented trunk stiffness with an orthosis could lead to a slightly reduced trunk muscle co-contraction, which may represent only a few percents of maximum voluntary activation [8,28], but still could compromise spine stability and lead to injury [29]. Furthermore, higher spine stiffness and lower muscle co-activation could obviate the need for active muscle reflex response to trunk perturbations as suggested by Stokes et al [30]. These neuromuscular changes would predispose an individual to low back injury or pain recurrence when sudden, unexpected loads on the spine occur [31,32]. Therefore, the purpose of this study was to document trunk stiffness and damping, as well as resultant changes in the muscle activation pattern and spine compression forces in healthy subjects during a 3-week period while they wore a LSO.

## Methods

### Subjects

Fourteen subjects (11 males and 3 females) volunteered for the study. Their age, weight, and height were on average (SD) 26 (8) yrs, 81(14) kg, and 180(13) cm, respectively. None of the subjects had any history of low back pain or neurophysiological disorders. At the beginning of the study, all subjects read and signed an informed consent form describing the experimental protocol that was approved by Yale University's Human Investigations Committee.

### Protocol

**Figure 1:**
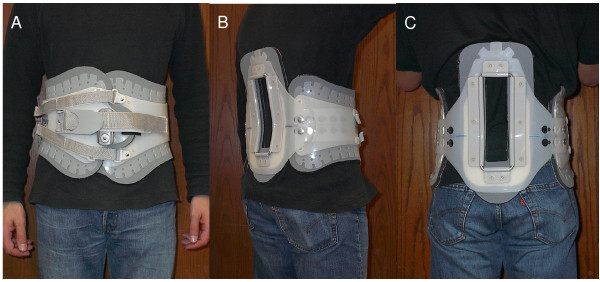
**Anterior (A), lateral (B), and posterior (C) views of the LSO used in the present study**.

The study protocol required all 14 subjects to wear a LSO (Aspen Medical Products, Inc., Long Beach, CA, USA) (Figure 1) for a period of 3 weeks. This device restricted trunk range of motion and increased trunk stiffness by amounts similar to comparable orthoses on the market, but was rated significantly more comfortable to wear by subjects [4]. Per manufacturer recommendations, the back panel of each individual LSO was adjusted to fit the contour of each subject's lumbar lordosis. Subjects were instructed to wear the LSO for a minimum of 3 hours a day during periods of activity. Any period sitting or lying down while wearing the LSO did not count towards the 3-hour daily minimum. This 3-hour period did not need to be continuous. Our preliminary studies suggested that healthy subjects might not comply with wearing a LSO for a longer period. To further maximize compliance, subjects were required to maintain a daily log in which they recorded the times they started and stopped wearing the LSO. In addition, one member of the research staff contacted each subject daily by telephone, email, or in person to verify that the LSO was worn. All subjects were instructed on how to wear and tighten the LSO. Initially, the LSO tension was adjusted to reach a pressure of 35 mmHg (4.7 kPa, measured with the Therapoint pressure measurement system, Roho, Inc., Belleville, IL, USA) between the LSO and abdominal wall at the location just lateral (left or right) to the umbilicus. This pressure was empirically selected when a balance between maximum tension in the brace and comfort to the user was achieved. The subjects then positioned and tightened their LSOs each day during the study to approximate that tension. The pressure measurement was set to 35 mmHg at the beginning of each testing session to standardize the LSO tension for all subjects.

The measurement of dependent variables was performed in three testing sessions on days 0, 7, and 21, with and without the LSO in each session. Half of the subjects were randomly selected to begin the testing sessions wearing the LSO, and the other half began their sessions without the LSO. This sequence was reversed at each subsequent testing session to correct for the effect of testing order.

### Tasks

**Figure 2:**
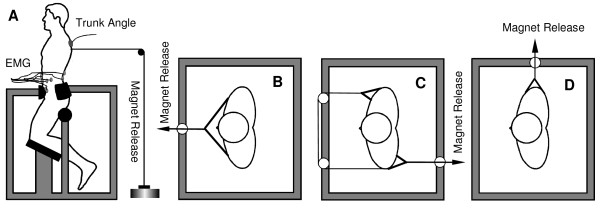
**The apparatus for a quick force release experiment**. The electromagnet release assembly could be moved to various points around the frame so that the quick force release testing trials could be conducted for trunk flexion (A), extension (B), right axial rotation (C), and left lateral bending (D).

Each testing session consisted of a quick trunk force release, isometric lift, and unsupported sitting tasks. For the quick force release task, each subject was placed in a semi-seated position in a custom-built apparatus and exerted isometric exertions in trunk flexion, extension, left lateral bending, and right axial rotation (Figure 2). This apparatus restrained lower body motion, leaving the upper body free to move in any direction. The pelvis was fixed at 4 points: the acetabulum via fixed femurs, the ischium, and at the anterior and posterior superior iliac spines. Thus, any postural adjustments through hip, knee, or ankle joints were eliminated. A cable attached to a chest harness at approximately the T5 level was held with an electromagnet and served as a resisting force. The subject pulled against the cable until (s)he reached a target force, displayed on an oscilloscope. As in our previous studies, the target force was set at 115 N for men and 80 N for women. Upon release, these forces resulted in the largest possible trunk displacement without being physically uncomfortable to the subjects [31,32]. An experimenter randomly released the electromagnet within 0 to 5 seconds after the target force was reached. The electromyographic (EMG) and trunk kinematics data were collected for 1 second prior to and 2 seconds after the release.

The isometric lifting task was performed by the subject while standing with slightly bent knees and the trunk flexed at the hips at 45 degrees with respect to the horizontal plane. A subject held 30% of his/her body mass in a crate with straight arms perpendicular to the floor. Trunk angle was measured with an inclinometer and the subject was instructed to maintain the natural lordotic curvature of the lumbar spine during the trial.

The sitting task was executed on a hard-surface seat with foot support but no back support. The knees formed a 90-degree angle and the arms were crossed on the chest. For both lifting and sitting tasks, EMG data were collected for 3 seconds after a correct, stable posture was achieved. All trials were repeated three times and the results were averaged across the three trials.

For the purpose of EMG normalization in each testing session, a series of maximum voluntary exertions (MVE) was executed, which were designed to maximally activate the latissimus dorsi and other trunk muscles while the subject attempted to perform sit-up, trunk extension, and lateral bending exercises [33]. These tasks were performed on the examination table with the experimenter providing manual resistance. Each MVE was sustained for 3 seconds.

### Data collection

EMG data from twelve major trunk muscles were recorded using bipolar, Ag-AgCl, disposable, surface electrodes (Graphic Controls, Buffalo, NY, USA). The electrodes were placed with center-to-center spacing of 3.5 cm over the following muscles on each side of the body: rectus abdominis (3 cm lateral to the umbilicus), external oblique (15 cm lateral to the umbilicus), internal oblique (approximately midway between the anterior superior iliac spine and symphysis pubis, above the inguinal ligament), latissimus dorsi (lateral to T9 over the muscle belly), thoracic erector spinae (5 cm lateral to T9 spinous process), and lumbar erector spinae (3 cm lateral to L3 spinous process). The EMG data recorded from these sites have previously been shown to be unaffected by the additional tension from wearing a LSO [34]. All EMG signals were band-pass limited between 20 and 420 Hz, differentially amplified (input impedance = 100 GΩ, CMRR > 140 dB), and sampled at 1600 Hz. The cardiac QRS waves were removed using the modified turning point and adaptive sampling algorithm [35].

The trunk kinematic response to the quick force release was measured with a three-dimensional electromagnetic motion-measurement device (Flock of Birds, Ascension Technology, Corp., Burlington, VT, USA). The source of a magnetic field was mounted on the testing apparatus and the sensor was attached to the subject at the T5 level using elastic straps. Angular displacements of the trunk were recorded at 120 Hz. All recorded data were processed to yield the following outcome measures: muscle response to the quick trunk force release, effective trunk stiffness, and spine compression force as a measure of total muscle activity.

### Muscle response to the quick force release

As shown in our previous work, following the quick force release, agonistic muscles that were active before the release were expected to shut-off reflexively. The antagonistic muscles that were inactive before the force release were expected to switch-on reflexively [36,37]. In flexion, trunk flexors acted as agonists and extensors as antagonists. In extension, trunk extensors acted as agonists and flexors as antagonists. In lateral bending to the left, ipsilateral muscles (left side) acted as agonists and contralateral muscles (right side) acted as antagonists. In right axial rotation, the right internal oblique, left external oblique, and left lumbar erector spinae muscles were considered agonists and the left internal oblique, right external oblique, and right lumbar erector spinae muscles were considered antagonists. The activation of the rectus abdominus, latissimus dorsi, and thoracic erector spinae were not considered in axial rotation because of the difficulty in classifying them functionally as agonists or antagonists.

The detection of onset and offset of muscle activities was automated using a model-based algorithm developed by Staude and Wolf [38] and implemented in the Matlab software environment (The MathWorks, Inc., Natick, MA, USA). This algorithm showed superior performance when compared to the traditional threshold based methods [38,39]. The EMG signal was first pre-processed with an adaptive whitening filter. The potential onsets and offsets were detected when significant changes occurred in the EMG signal modeled as a Gaussian random process. All such detected events were then ranked based on their maximum likelihood statistics. An appropriate event with the largest generalized likelihood ratio was selected as the onset or offset from the 20 to 150 ms time interval following the force release. The assumption was made that reflex responses could not occur any earlier than 20 ms after the stimulus. Any responses occurring later than 150 ms after the stimulus may represent voluntary and not reflexive muscle activity.

To quantify the overall trunk muscles' response to the quick force release, the average latency and the number of muscles that responded to the quick force release were computed as in our previous studies [36,37]. There were six muscles expected to switch-on and six to shut-off in the trunk flexion, extension, and lateral bending trials. Thus, the dependent measures in the quick force release trials were (i) average latency and (ii) the number of the muscles switching-on and shutting-off in each direction of the quick force release. A high background muscle activity in axial rotation precluded the reliable identification of muscle onsets and offsets in that quick force release direction.

### Effective trunk stiffness and damping

The effective trunk stiffness was estimated from the trunk kinematic response to the quick force release [6]. The trunk was represented with a second order, inverted pendulum model. Its angular displacement response (θ) to the force release is determined by the trunk stiffness (K), inertia (I), and damping (B) (Equation 1):(1)(2)

where C is a constant related to force, mg is trunk weight, and L is the height measured from the L4-L5 joint to the center of trunk mass, assumed to be at the T9 level. Muscle reflex response modifies the trunk kinematics after the release [40]. Therefore, the stiffness (K) and damping (B) are called the "effective trunk stiffness" and "effective trunk damping", respectively, because they combine the effects of the trunk muscles' stiffness and damping established prior to the release and the muscle reflex response following the release [40]. The effective trunk stiffness and damping was obtained from fitting the parameters B, K, and C in the Equation 2 to the experimental data (θ) up to the maximum trunk displacement, according to the previously established method [6,40]. The inertia (I) was computed as 53.6% of body weight times L^2 ^[41].

### Spine compression force

The spine compression force reflected the overall trunk muscle activation level established immediately prior to the quick force release. The estimate of the spine compression force was obtained from a detailed biomechanical model of the lumbar spine, which we have described earlier [29]. The model consisted of five lumbar vertebrae between the rigid pelvis and ribcage, and 90 muscle fascicles. Each intervertebral joint was represented by a nonlinear, lumped parameter disc and ligament equivalent for stiffness about the three axes of rotation. Thus, the system consisted of 18 degrees of freedom (6 joints × 3 degrees of freedom each).

Muscle forces were estimated based on 200 ms of EMG data recorded immediately before the force release from 12 trunk muscles. The EMG data were rectified, averaged, and expressed in %MVE. After accounting for the contributions of passive tissues, the moments and forces necessary to balance external loads and upper body weight were distributed among all 90 muscle fascicles using an EMG-assisted optimization method [42]. The muscle forces and muscle stiffness were first estimated from EMG data using a cross-bridge bond distribution moment model reflecting muscle contraction dynamics [43]. A quadratic optimization algorithm was subsequently applied to minimize the adjustment of individual muscle forces (muscle gains), while requiring that the moment equations about the three rotational axes of every intervertebral joint were balanced [44]. All muscle forces and external loads were then summed along the axis perpendicular to the L4-L5 intervertebral disc to obtain a joint compression force. The direct effects of intra-abdominal pressure or the 35 mmHg LSO pressure on spine compression force were not considered.

### Statistical analysis

A three-factor, repeated-measures ANOVA and a Tukey's post-hoc test were used to examine the effects of the LSO condition (LSO), testing session (Session), and the direction of exertion (Direction) on all of the dependent variables. A Box-Cox transformation was applied to any data which did not follow a normal distribution. A non-parametric, Kruskal-Wallis test was used to compare the number of muscles responding to the quick force release, because these data could not be corrected for normality. The significance level was set at *P *= 0.05. All statistical analyses were performed with the Minitab 13.1 statistical software (Minitab Inc., State College, PA, USA).

## Results

Based on their daily logs and personal communication with researchers, all subjects adhered to the prescribed experimental protocol for wearing the LSO. On average, the subjects wore their LSOs for 3.1 hrs/day (SD = 1.1). There was no difference in the cumulative duration of the LSO wear between each week (df = 2, F = 0.38, *P *= 0.69).

### Muscle response to the quick force release

The only significant main effect in the trunk muscle response latencies to the quick force release was due to the direction of the force release (Table 1). On average, the muscle offset latencies were shorter in trunk extension or flexion than in lateral bending (67(35) and 67(31) vs. 78(35) ms, respectively) (df = 2; F = 3.78; *P *= 0.024). The average muscle onset latencies were shortest in trunk extension, then in flexion, and the longest in lateral bending (55(7), 60(10), and 64(10) ms, respectively) (df = 2; F = 25.85; *P *< 0.001). There were no other main effects or their interactions present in the muscle reflex latencies involving the LSO or Session (*P *> 0.5).

**Table 1:** Average (standard deviations in parenthesis) latencies of trunk muscle responses to quick force release.

	Muscle Offset Latencies [ms]					
	
	Extension		Flexion		**Lateral Bending **^**#**^	
	
	No LSO	LSO	No LSO	LSO	No LSO	LSO
Day 0	51 (27)	71 (35)	72 (40)	63 (30)	75 (33)	73 (38)
Day 7	77 (49)	66 (31)	59 (28)	76 (30)	81 (37)	85 (38)
Day 21	66 (36)	68 (25)	74 (36)	60 (24)	86 (40)	68 (24)
Average	65 (39)	68 (30)	68 (34)	67 (29)	81 (36)	75 (34)
						
	**Muscle Offset Latencies [ms]**					
	
	**Extension **^**#**^		**Flexion **^**#**^		**Lateral Bending **^**#**^	
	
	**No LSO**	**LSO**	**No LSO**	**LSO**	**No LSO**	**LSO**

Day 0	56 (8)	55 (6)	58 (8)	62 (10)	62 (9)	65 (10)
Day 7	57 (9)	55 (6)	60 (9)	63 (8)	63 (10)	67 (10)
Day 21	55 (7)	55 (6)	59 (13)	61 (9)	64 (13)	63 (7)
Average	56 (8)	55 (6)	59 (10)	62 (9)	63 (10)	65 (9)

^**# **^denotes a statistical significance at *P *< 0.05 level.

The average number of agonist muscles switching off following the quick force release was significantly greater when the LSO was worn compared to the No LSO condition (1.8(1.0) LSO vs. 1.4(1.0) No LSO) (df = 1, H = 8.58, *P *= 0.003) (Table 2). On the other hand, the number of antagonists responding with an onset of muscle activity was significantly greater on the 2^nd ^test (day 7) (df = 2; H = 8.64; *P *= 0.013) (Table 2). No other main effects were present in the number of muscles responding to the quick force release.

**Table 2:** Average (standard deviations in parenthesis) number of muscles for which a response to quick force release was detected.

	Number of Muscles Shutting Off					
	
	Extension		Flexion		Lateral Bending	
	
	**No LSO **^**#**^	**LSO **^**#**^	**No LSO **^**#**^	**LSO **^**#**^	**No LSO **^**#**^	**LSO **^**#**^
Day 0	1.6 (0.8)	1.6 (0.6)	1.3 (1.1)	1.6 (1.4)	0.9 (0.6)	1.6 (0.8)
Day 7	1.5 (0.8)	1.8 (0.9)	1.7 (1.5)	1.8 (1.1)	1.2 (0.7)	1.9 (1.0)
Day 21	1.8 (1.1)	2.2 (0.8)	1.6 (1.0)	2.0 (1.1)	1.5 (0.7)	1.4 (0.9)
Average	1.6 (0.9)	1.9 (0.8)	1.5 (1.2)	1.8 (1.2)	1.2 (0.7)	1.6 (0.9)
						
	**Number of Muscles Shutting On**					
	
	**Extension**		**Flexion**		**Lateral Bending**	
	
	**No LSO**	**LSO**	**No LSO**	**LSO**	**No LSO**	**LSO**

Day 0	5.3 (1.6)	5.7 (0.3)	4.7 (1.5)	4.8 (0.7)	4.8 (1.5)	5.2 (0.6)
Day 7 ^**#**^	5.9 (0.2)	6.0 (0.1)	4.8 (1.7)	5.1 (0.7)	5.2 (0.8)	5.0 (0.8)
Day 21	5.6 (0.6)	5.5 (0.5)	5.0 (0.8)	5.0 (0.6)	4.9 (0.9)	4.6 (1.0)
Average	5.6 (1.0)	5.7 (0.4)	4.9 (1.3)	5.0 (0.7)	5.0 (1.1)	4.9 (0.8)

^**# **^denotes a statistical significance at *P *< 0.05 level.

### Effective trunk stiffness and damping

**Figure 3:**
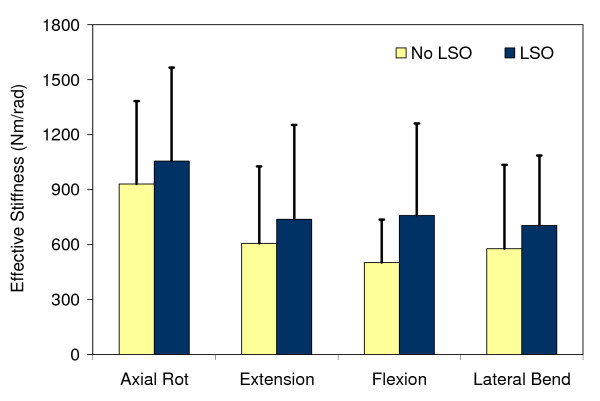
**Effective trunk stiffness obtained from trunk kinematics in the quick force release experiment and averaged by week**. Bars represent one standard deviation.

The LSO significantly (df = 1; F = 17.21; *P *< 0.001) increased the effective trunk stiffness by 160 Nm/rad or 27% when averaged across all directions and testing sessions (Figure 3). The testing direction also had a significant effect on trunk stiffness (df = 3; F = 20.85; *P *< 0.001), but no statistical interactions with the other factors of interest (i.e. LSO, Session) were found. There was no significant change over time in the effective trunk stiffness estimated from the trunk kinematic response to the quick force release (*P *> 0.05).

Similar to stiffness, the LSO increased significantly the effective trunk damping by 4.4 Ns/rad or 12%, on average, across all sessions and directions (df = 1, F = 4.22, *P *= 0.04). There was also a significant effect of direction, for which the effective trunk damping ranged from 33.4 (26.1) Ns/rad in extension to 47.8 (36.0) Ns/rad in axial rotation.

### Spine compression force

**Figure 4:**
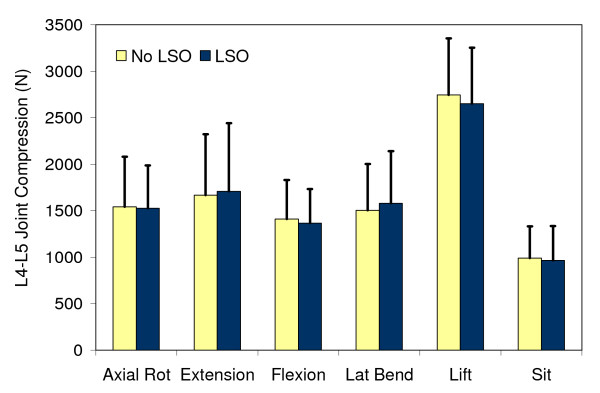
**Spine compression force at the L4-L5 joint estimated from the isometric trunk exertions just prior to the quick force release**. The data were averaged across all quick force release trials by week. Bars represent one standard deviation.

The spine compression force reflected the aggregate trunk muscle activation during the isometric exertions just prior to the quick force release, and during the sitting and lifting tasks. No statistically significant effects of the LSO or Session were present (*P *> 0.05) (Figure 4). The only significant differences in spine compression force were due to the different tasks and the direction of the quick force release (Figure 4).

## Discussion

Changes in trunk muscle activation due to wearing the LSO over a three-week period were studied using quick force release experiments and common tasks such as sitting and lifting. Each of the outcome variables was measured independently and carried slightly different information about trunk muscle function. Because trunk moments were precisely controlled in all of the tasks, any changes in the measured variables would reflect the neuromuscular changes in motor control of the trunk. The muscle response latencies described the reflexive muscle function in response to sudden loading/unloading of the spine. Spine compression force reflected the changes in the overall magnitude of agonist and antagonist muscle co-activation during isometric trunk exertions prior to the quick force release. Finally, the effective trunk stiffness and damping combined the effects of these properties established prior to the quick force release and muscle reflex response after the quick force release into an overall measure of trunk impedance to perturbations [40].

The testing session did not significantly affect any of the measured variables, except for a larger number of muscles switching on in response to the quick force release on day 7. However, on average, this difference was only 0.2 muscles between days 0 and 7 and it disappeared by day 21 (Table 2). This finding is likely not clinically relevant. Therefore, it should be concluded that wearing a LSO for 3 weeks, 3 hours a day by healthy subjects did not result in detrimental changes to the neuromuscular function of trunk muscles.

Another finding, the increased trunk stiffness provided by the LSO, was expected and consistent with previous studies [5-7,23,45]. However, to our knowledge, there has been no report of a significantly greater number of agonist muscles shutting-off in response to the quick force release when the LSO was worn. This finding was consistent across all directions and was similar for all testing sessions, suggesting that it is an effect of the LSO alone and not related to wearing a LSO for an extended period.

The effects of lumbar orthoses on muscle reflex response to sudden trunk perturbations were studied in the past only by Lavender et al. [7] and Pfeifer et al. [46]. Pfeifer et al. reported that the left erector spinae muscle responded later and the obliquus abdominis responded sooner when the subjects wore lumbar orthoses and were struck unexpectedly from behind with a pendulum [46]. Furthermore, these reflex responses were of smaller amplitude in the erector spinae and the right obliquus abdominis muscles when the orthoses were worn. In contrast, Lavender et al. found the greater peak muscle response in the left latissimus dorsi, erector spinae, and external oblique; and the smaller peak response in right external oblique, rectus abdominis, and the left erector spinae in asymmetric sudden trunk loading when the belt was tensioned [7]. These findings along with our study suggest that some neuromuscular adaptations occur immediately when wearing the LSO.

Such neuromuscular adaptations could be the result of increased trunk stiffness and the cutaneous stimulation derived from the LSO. Passively increased trunk stiffness with a LSO impairs postural control of the trunk [47] and may require different feedback gains if the original level of performance is required [8,28]. In addition, it is well documented that cutaneous afferents can influence the gamma fusimotor activity in muscle spindles [48-50] and may alter trunk proprioception when a LSO is worn [51]. All of these mechanisms lead to highly variable neuromuscular adaptations and their functional implications are difficult to interpret at this time. Such different changes in the muscle activation patterns are not surprising [52] and more research is necessary to formulate practical conclusions and recommendations regarding the long-term usage of LSOs. For now, it appears that wearing the LSO for 3 weeks, 3 hours a day does not produce any significant changes in trunk muscle function.

There are several limitations to this study. Wearing a LSO 3 hrs a day for 21 days may not have been enough time for significant neuromuscular changes to occur. The results may have been different if another orthosis was used. However, the Aspen LSO provides similar biomechanical effects to other popular orthoses on the market and is therefore representative of commonly used devices [1-7,23,45]. Deep trunk muscles were not monitored with EMG in the current study. It is possible that activities of these muscles changed in response to wearing a LSO and they contributed to the significant increase in trunk stiffness when the LSO was worn. Finally, only healthy, young subjects were tested, rendering the findings from this study less applicable to an older patient population with LBP.

It has been shown by several research groups that patients with LBP exhibit delayed muscle latencies in response to sudden trunk loading [36,37,53-55]. More importantly, these patients tend to shut-off a significantly smaller number of agonist muscles in response to the quick force release compared to healthy control subjects [36]. This suggests that the increased number of muscles shutting-off seen in our study, brought about by wearing the LSO, could lead to an improved neuromuscular feedback control of unexpected sudden spinal loading in patients with LBP. This could also explain the feeling of increased confidence reported by patients engaging in physical activity when wearing the LSO [9-12]. This hypothesis should be addressed in future studies involving patients with LBP.

## Conclusions

There were no clinically significant changes in neuromuscular control of the trunk during 3 weeks of wearing a LSO for 3 hours a day. The LSO increased trunk stiffness across all quick force release directions and testing sessions. Along with the increased effective trunk stiffness, the LSO brought about an increased number of agonist muscles shutting-off in response to the quick force release. Further research is needed to determine the clinical significance of such effects in individuals with LBP.

## Competing interests

The authors declare that they have no competing interests.

## Authors' contributions

JC designed and managed the study. KM and KS were responsible for data collection and analysis. JC and AL participated in data analysis and drafted the manuscript. All authors critically read, revised, and finally approved the manuscript.

## Pre-publication history

The pre-publication history for this paper can be accessed here:

http://www.biomedcentral.com/1471-2474/11/154/prepub

## References

[B1] Lantz SA Schultz AB Lumbar spine orthosis wearing. I. Restriction of gross body motions Spine 1986 11 8 834 837 10.1097/00007632-198610000-00019 3810301

[B2] Buchalter D Kahanovitz N Viola K Dorsky S Nordin M Three-dimensional spinal motion measurements. Part 2: A noninvasive assessment of lumbar brace immobilization of the spine J Spinal Disord 1988 1 4 284 286 10.1097/00002517-198800140-00001 2980256

[B3] van Poppel MN de Looze MP Koes BW Smid T Bouter LM Mechanisms of action of lumbar supports: a systematic review Spine 2000 25 16 2103 2113 10.1097/00007632-200008150-00016 10954643

[B4] Krag MH Fox MJ Haugh LD Comparison of three lumbar orthoses using motion assessment during task performance Spine 2003 28 20 2359 2367 10.1097/01.BRS.0000085328.71345.54 14560084

[B5] McGill S Seguin J Bennett G Passive stiffness of the lumbar torso in flexion, extension, lateral bending, and axial rotation. Effect of belt wearing and breath holding Spine 1994 19 6 696 704 10.1097/00007632-199403001-00009 8009335

[B6] Cholewicki J Juluru K Radebold A Panjabi MM McGill SM Lumbar spine stability can be augmented with an abdominal belt and/or increased intra-abdominal pressure Eur Spine J 1999 8 5 388 395 10.1007/s005860050192 10552322 PMC3611203

[B7] Lavender SA Shakeel K Andersson GB Thomas JS Effects of a lifting belt on spine moments and muscle recruitments after unexpected sudden loading Spine 2000 25 12 1569 1578 10.1097/00007632-200006150-00018 10851108

[B8] Cholewicki J The effects of lumbosacral orthoses on spine stability: what changes in EMG can be expected? J Orthop Res 2004 22 5 1150 1155 10.1016/j.orthres.2004.01.009 15304292

[B9] Ahlgren SA Hansen T The use of lumbosacral corsets prescribed for low back pain Prosthet Orthot Int 1978 2 2 101 104 10.3109/03093647809177777 152906

[B10] Million R Nilsen KH Jayson MI Baker RD Evaluation of low back pain and assessment of lumbar corsets with and without back supports Ann Rheum Dis 1981 40 5 449 454 10.1136/ard.40.5.449 6458250 PMC1000779

[B11] Alaranta H Hurri H Compliance and subjective relief by corset treatment in chronic low back pain Scand J Rehabil Med 1988 20 3 133 136 2973123

[B12] Jellema P Bierma-Zeinstra SM Van Poppel MN Bernsen RM Koes BW Feasibility of lumbar supports for home care workers with low back pain Occup Med (Lond) 2002 52 6 317 323 10.1093/occmed/52.6.317 12361993

[B13] Calmels P Queneau P Hamonet C Le Pen C Maurel F Lerouvreur C Thoumie P Effectiveness of a lumbar belt in subacute low back pain: an open, multicentric, and randomized clinical study Spine (Phila Pa 1976) 2009 34 3 215 220 19179915 10.1097/BRS.0b013e31819577dc

[B14] McGill SM Abdominal belts in industry: a position paper on their assets, liabilities and use Am Ind Hyg Assoc J 1993 54 12 752 754 8304280 10.1080/15298669391355332

[B15] Koes BW van den Hoogen HMM Efficacy of bed rest and orthoses on low back pain: a review of randomized clinical trials Eur J Phys Med Rehabil 1994 4 86 93

[B16] McGill SM Karwowski W, Marras WS Update on the use back belts in industry: more data - same conclusion The Occupational Ergonomics Handbook 1998 CRC Press 1353 1358

[B17] Jellema P van Tulder MW van Poppel MN Nachemson AL Bouter LM Lumbar supports for prevention and treatment of low back pain: a systematic review within the framework of the Cochrane Back Review Group Spine 2001 26 4 377 386 10.1097/00007632-200102150-00014 11224885

[B18] van Duijvenbode IC Jellema P van Poppel MN van Tulder MW Lumbar supports for prevention and treatment of low back pain Cochrane Database Syst Rev 2008 2 CD001823 18425875 10.1002/14651858.CD001823.pub3 PMC7046130

[B19] Bible JE Biswas D Whang PG Simpson AK Rechtine GR Grauer JN Postoperative bracing after spine surgery for degenerative conditions: a questionnaire study Spine J 2009 9 4 309 316 10.1016/j.spinee.2008.06.453 18790685

[B20] Phaner V Fayolle-Minon I Lequang B Valayer-Chaleat E Calmels P Are there indications (other than scoliosis) for rigid orthopaedic brace treatment in chronic, mechanical low back pain? Ann Phys Rehabil Med 2009 52 5 382 393 19524496 10.1016/j.rehab.2009.05.002

[B21] Eisinger DB Kumar R Woodrow R Effect of lumbar orthotics on trunk muscle strength Am J Phys Med Rehabil 1996 75 3 194 197 10.1097/00002060-199605000-00008 8663926

[B22] Rohlmann A Bergmann G Graichen F Neff G Braces do not reduce loads on internal spinal fixation devices Clin Biomech 1999 14 2 97 102 10.1016/S0268-0033(98)00056-4 10619096

[B23] Ivancic PC Cholewicki J Radebold A Effects of the abdominal belt on muscle-generated spinal stability and L4/L5 joint compression force Ergonomics 2002 45 7 501 513 10.1080/00140130210136035 12167204

[B24] Walsh NE Schwartz RK The influence of prophylactic orthoses on abdominal strength and low back injury in the workplace Am J Phys Med Rehabil 1990 69 5 245 250 10.1097/00002060-199010000-00004 2145877

[B25] Holmström E Moritz U Effects of lumbar belts on trunk muscle strength and endurance: a follow-up study of construction workers J Spinal Disord 1992 5 3 260 266 10.1097/00002517-199209000-00003 1387822

[B26] Fayolle-Minon I Calmels P Effect of wearing a lumbar orthosis on trunk muscles: study of the muscle strength after 21days of use on healthy subjects Joint Bone Spine 2008 75 1 58 63 10.1016/j.jbspin.2007.04.018 18029219

[B27] Reddell CR Congleton JJ Huchingson RD Montgomery JF An evaluation of a weightlifting belt and back injury prevention training class for airline baggage handlers Appl Ergon 1992 23 5 319 329 10.1016/0003-6870(92)90293-5 15676878

[B28] Cholewicki J Reeves NP Everding VQ Morrisette DC Lumbosacral orthoses reduce trunk muscle activity in a postural control task J Biomech 2007 40 8 1731 1736 10.1016/j.jbiomech.2006.08.005 17054963

[B29] Cholewicki J McGill SM Mechanical stability of the in vivo lumbar spine: implications for injury and chronic low back pain Clin Biomech 1996 11 1 1 15 10.1016/0268-0033(95)00035-6 11415593

[B30] Stokes IAF Gardner-Morse M Henry SM Badger GJ Decrease in trunk muscular response to perturbation with preactivation of lumbar spinal musculature Spine 2000 25 15 1957 1964 10.1097/00007632-200008010-00015 10908940

[B31] Cholewicki J Silfies SP Shah RA Greene HS Reeves NP Alvi K Goldberg B Delayed trunk muscle reflex responses increase the risk of low back injuries Spine 2005 30 23 2614 2620 10.1097/01.brs.0000188273.27463.bc 16319747

[B32] Hodges P van den Hoorn W Dawson A Cholewicki J Changes in the mechanical properties of the trunk in low back pain may be associated with recurrence J Biomech 2009 42 1 61 66 10.1016/j.jbiomech.2008.10.001 19062020

[B33] McGill SM Electromyographic activity of the abdominal and low back musculature during the generation of isometric and dynamic axial trunk torque: implications for lumbar mechanics J Orthop Res 1991 9 1 91 103 10.1002/jor.1100090112 1824571

[B34] Jorgensen MJ Marras WS The effect of lumbar back support tension on trunk muscle activity Clin Biomech 2000 15 4 292 294 10.1016/S0268-0033(99)00067-4 10675671

[B35] Cholewicki J Panjabi MM Khachatryan A Stabilizing function of trunk flexor-extensor muscles around a neutral spine posture Spine 1997 22 19 2207 2212 10.1097/00007632-199710010-00003 9346140

[B36] Radebold A Cholewicki J Panjabi MM Patel TC Muscle response pattern to sudden trunk loading in healthy individuals and in patients with chronic low back pain Spine 2000 25 8 947 954 10.1097/00007632-200004150-00009 10767807

[B37] Cholewicki J Polzhofer GK Galloway MT Greene HS Shah RA Radebold A Neuromuscular function in athletes following recovery from an acute low back injury J Orthop Sports Phys Ther 2002 32 568 575 12449256 10.2519/jospt.2002.32.11.568

[B38] Staude G Wolf W Objective motor response onset detection in surface myoelectric signals Med Eng Phys 1999 21 6-7 449 467 10.1016/S1350-4533(99)00067-3 10624741

[B39] Lee AS Cholewicki J Reeves NP The effect of background muscle activity on computerized detection of sEMG onset and offset J Biomech 2007 40 15 3521 3526 10.1016/j.jbiomech.2007.05.012 17588589 PMC2098870

[B40] Cholewicki J Simons APD Radebold A Effects of external trunk loads on lumbar spine stability J Biomech 2000 33 11 1377 1385 10.1016/S0021-9290(00)00118-4 10940396

[B41] Winter DA Biomechanics and Motor Control of Human Movement 2005 3 New York, NY: John Wiley & Sons

[B42] Cholewicki J McGill SM Norman RW Comparison of muscle forces and joint load from an optimization and EMG assisted lumbar spine model: towards development of a hybrid approach J Biomech 1995 28 3 321 331 10.1016/0021-9290(94)00065-C 7730390

[B43] Cholewicki J McGill SM Relationship between muscle force and stiffness in the whole mammalian muscle: a simulation study J Biomech Eng 1995 117 3 339 342 10.1115/1.2794189 8618387

[B44] Cholewicki J McGill SM EMG assisted optimization: a hybrid approach for estimating muscle forces in an indeterminate biomechanical model J Biomech 1994 27 10 1287 1289 10.1016/0021-9290(94)90282-8 7962016

[B45] Cholewicki J Alvi K Silfies SP Bartolomei J Comparison of motion restriction and trunk stiffness provided by three thoraco-lumbo-sacral orthoses (TLSOs) J Spinal Disord Tech 2003 16 5 461 468 10.1097/00024720-200310000-00005 14526195

[B46] Pfeifer K Vogt L Klingler J Portscher M Banzer W Sensomotor function while wearing lumbar corsets Z Orthop Ihre Grenzgeb 2001 139 1 12 18 10.1055/s-2001-11865 11253516

[B47] Reeves NP Everding VQ Cholewicki J Morrisette DC The effects of trunk stiffness on postural control during unstable seated balance Exp Brain Res 2006 174 4 694 700 10.1007/s00221-006-0516-5 16724177

[B48] Johansson H Sjölander P Sojka P Wadell I Effects of electrical and natural stimulation of skin afferents on the gamma-spindle system of the triceps surae muscle Neurosci Res 1989 6 6 537 555 10.1016/0168-0102(89)90043-6 2797506

[B49] Ellaway PH Davey NJ Ferrell WR Baxendale RH The action of knee joint afferents and the concomitant influence of cutaneous (sural) afferents on the discharge of triceps surae gamma- motoneurones in the cat Exp Physiol 1996 81 1 45 66 8869139 10.1113/expphysiol.1996.sp003918

[B50] Rudomin P Selectivity of the central control of sensory information in the mammalian spinal cord Adv Exp Med Biol 2002 508 157 170 12171106 10.1007/978-1-4615-0713-0_19

[B51] Cholewicki J Shah KR McGill KC The effects of a 3-week use of lumbosacral orthoses on proprioception in the lumbar spine J Orthop Sports Phys Ther 2006 36 4 225 231 16676872 10.2519/jospt.2006.36.4.225

[B52] van Dieën JH Selen LP Cholewicki J Trunk muscle activation in low-back pain patients, an analysis of the literature J Electromyogr Kinesiol 2003 13 4 333 351 10.1016/S1050-6411(03)00041-5 12832164

[B53] Magnusson ML Aleksiev A Wilder DG Pope MH Spratt K Lee SH Goel VK Weinstein JN Unexpected load and asymmetric posture as etiologic factors in low back pain Eur Spine J 1996 5 1 23 35 10.1007/BF00307824 8689414

[B54] Hodges PW Richardson CA Inefficient muscular stabilization of the lumbar spine associated with low back pain. A motor control evaluation of transversus abdominis Spine 1996 21 22 2640 2650 10.1097/00007632-199611150-00014 8961451

[B55] Hodges PW Richardson CA Delayed postural contraction of transversus abdominis in low back pain associated with movement of the lower limb J Spinal Disord 1998 11 1 46 56 10.1097/00002517-199802000-00008 9493770

